# Prokineticin receptor-1-dependent paracrine and autocrine pathways control cardiac tcf21^+^ fibroblast progenitor cell transformation into adipocytes and vascular cells

**DOI:** 10.1038/s41598-017-13198-2

**Published:** 2017-10-16

**Authors:** Rehana Qureshi, Michel Kindo, Himanshu Arora, Mounia Boulberdaa, Marja Steenman, Canan G. Nebigil

**Affiliations:** 1 University of Strasbourg, CNRS, Biotechnology and Cell Signaling Laboratory (UMR 7242), Illkirch, France; 2 Hospital of University of Strasbourg, Cardiovascular surgery department, Strasbourg, France; 3 Institute of Thorax, INSERM UMR 1087/CNRS UMR 6291, Nantes, France

## Abstract

Cardiac fat tissue volume and vascular dysfunction are strongly associated, accounting for overall body mass. Despite its pathophysiological significance, the origin and autocrine/paracrine pathways that regulate cardiac fat tissue and vascular network formation are unclear. We hypothesize that adipocytes and vasculogenic cells in adult mice hearts may share a common cardiac cells that could transform into adipocytes or vascular lineages, depending on the paracrine and autocrine stimuli. In this study utilizing transgenic mice overexpressing prokineticin receptor (PKR1) in cardiomyocytes, and tcf21ERT-cre^TM^-derived cardiac fibroblast progenitor (CFP)-specific PKR1 knockout mice (PKR1^*tcf*−/−^), as well as FACS-isolated CFPs, we showed that adipogenesis and vasculogenesis share a common CFPs originating from the tcf21^+^ epithelial lineage. We found that prokineticin-2 is a cardiomyocyte secretome that controls CFP transformation into adipocytes and vasculogenic cells *in vivo* and *in vitro*. Upon HFD exposure, PKR1^*tcf*−/−^ mice displayed excessive fat deposition in the atrioventricular groove, perivascular area, and pericardium, which was accompanied by an impaired vascular network and cardiac dysfunction. This study contributes to the cardio-obesity field by demonstrating that PKR1 via autocrine/paracrine pathways controls CFP–vasculogenic- and CFP-adipocyte-transformation in adult heart. Our study may open up new possibilities for the treatment of metabolic cardiac diseases and atherosclerosis.

## Introduction

Cardiac fibroblast and progenitors (CFPs) derived from epicardium and endothelium respond to a wide range of different stimuli, including hypoxia as well as chemical, mechanical, and electrical signals, during cardiac development and disease^1^. The majority of CFPs derived from epicardium expresses the basic helix-loop-helix (bHLH) transcription factor tcf21 (pod1/epicardin/capsulin), which is similar to Wt1 and Tbx18, in the developing embryonic heart. However, tcf21 continues to be expressed within resting CFPs in the adult heart^2^, which represent the primary progenitor pool in the adult heart^3,4^. Tcf21-expressing cells populate areas of the epicardium or the perivascular or interstitial areas, depending on the injury type^5^. The activated CFPs might have reparative functions after environmental changes. Whether cardiac tcf21^+^ CFPs undergo adipocyte or vascular-transformations in the adult heart depending on the stimuli and different environmental contexts has not yet been studied. Hence, cardiomyocyte-derived paracrine pathways that regulate the differentiation of CFPs remain to be studied.

Prokineticin-2 is an anorexigenic and angiogenic hormone that acts through two G protein-coupled receptors, prokineticin receptor (PKR) 1 and PKR2^6^. The absence of PKR1 in adipose tissue causes an increase in adipose tissue mass, leading to obesity^7^. Prokineticin-2/PKR1 signaling inhibits the differentiation of adipocyte progenitor cells into adipocytes. It also promotes epithelial-to-mesenchymal transition (EMT) in the epicardial Wt1^+^lineage during the development of coronary vasculature and growth of the ventricular wall^8^. Prokineticin-2, via PKR1, induces the differentiation of adult epicardial explant cultures into endothelial and vascular cells^9^. A transgenic mouse model in which PKR1 is overexpressed in the cardiomyocytes (TG-PKR1) has shown that PKR1 signaling upregulates the expression of its own ligand, prokineticin-2, as a secretome, inducing the proliferation and differentiation of epicardial progenitor cells (EPDCs), thereby promoting neovascularization^9^.

Here, we examined whether PKR1-dependent cardiomyocyte signaling is involved in a novel biological event. We utilized transgenic mice overexpressing PKR1 under the regulation of cardiomyocyte-specific alpha myosin heavy chain (αMHC) (TG-PKR1) and an *in vitro* cardiac cell system. To study whether adipocytes and vasculogenic cells share common epicardial-derived CFPs, we generated CFP-specific PKR1 knockout mice (PKR1^*tcf*−/−^) utilizing the conditional tcf21 cre^Tomato^-driver mice and isolated Tomato/tcf21-positive CFPs by fluorescence-activated cell sorting (FACS). We identified a novel role for PKR1 signaling in regulating cardiac fat tissue and vascular network development, suggesting that defective PKR1 signaling in obesity could be a key contributor to metabolic cardiac diseases and atherosclerosis.

## Results

### TG-PKR1 hearts exhibit low levels of PPARγ gene expression

**Figure 1:**
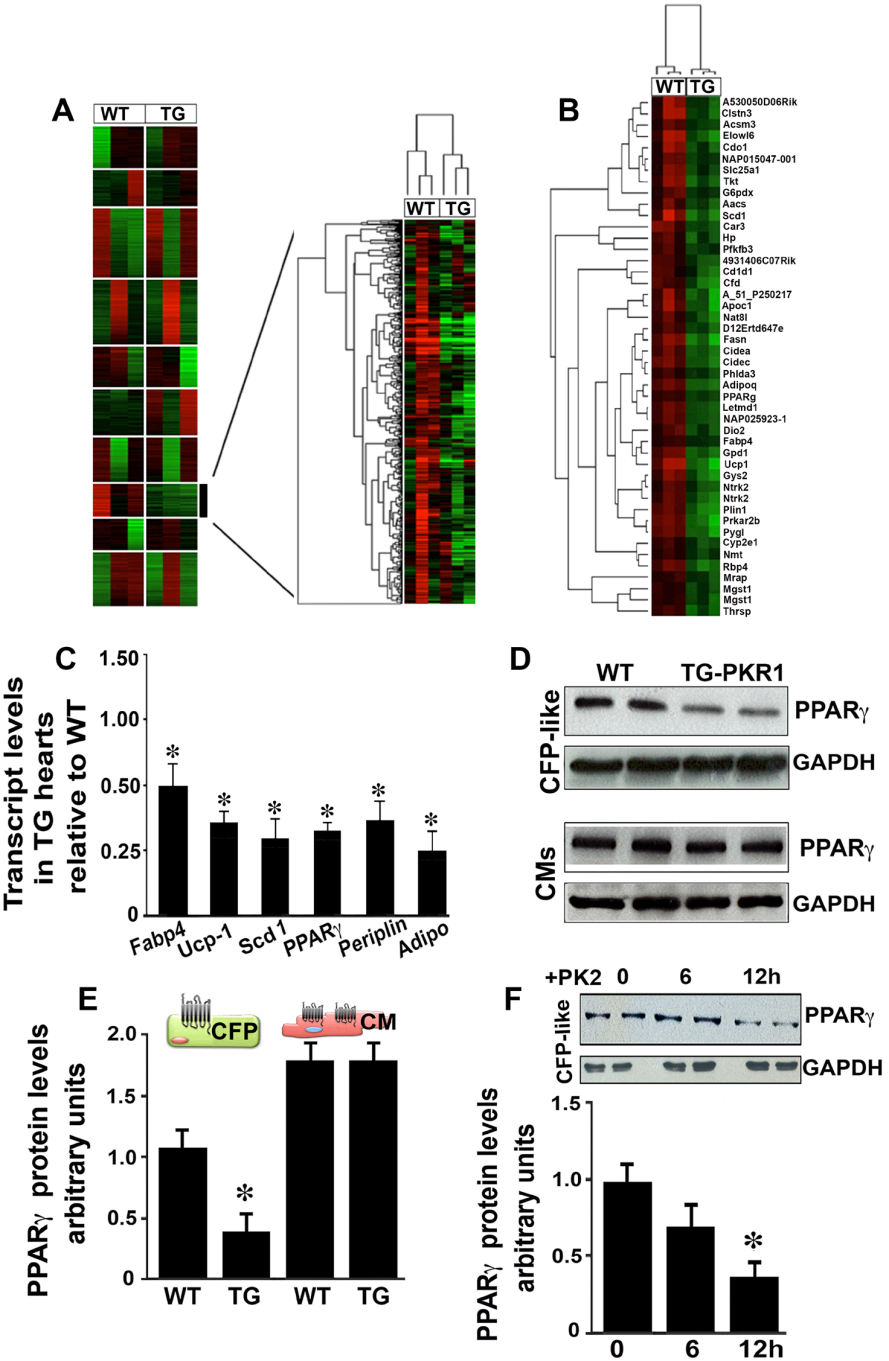
Microarray analyses of TG-PKR1 hearts. (**A**) K-means analysis of the microarray data: gene expression is presented as a colored matrix, where each row represents a gene and each column represents a sample. Green, black and red correspond to lower values, median values and higher values, respectively. Left: transcriptome data clustered by K-means (with k = 10). The highlighted cluster 8 clearly distinguishes WT from TG-PKR1 mice (shown by hierarchical clustering on the right). (**B**) Hierarchical clustering of the samples based on the 46 expression profiles of WT and TG-PKR1 mice (12 weeks old). All n = 3 mice/group. (**C**) qPCR analyses of the PPARγ signaling pathway-related genes (*Fabp4*, *UCP-1*, *Scd1*, *PPARγ*, *perilipin* and *adiponectin*) involved in adipogenesis (*p < 0.05, n = 4; *t-test*). (**D**) Western blot analyses of PPARγ protein levels in cultured CF-like non-cardiomyocytes and cardiomyocytes derived from TG-PKR1 and WT hearts. (**E**) Histogram shows the quantification of PPARγ protein levels in the CF-like cells and cardiomyocytes (*p < 0.05, n = 4; *two way ANOVA*). (**F**) Western blot analyses of PPARγ protein levels in CF-like cells upon prokineticin-2 (PK2) treatment at the indicated times. Histogram shows the quantification of PPARγ protein levels after PK2 exposure in the CF-like cells (*p < 0.05, n = 3; *two way ANOVA*). All n = 6 mice/group. Original blots are shown in Figure S5.

To explore novel cardiac events involved in signaling from cardiomyocytes to non-cardiomyocytes, we first performed microarray analyses to compare the gene expression profiles of the hearts from wild type (WT) and TG-PKR1 mice; differential expression of PKR1-regulated genes was observed. K-means analysis identified one cluster of genes with differentially expressed levels between the two groups (Fig. 1A). The genes in this cluster were all expressed at lower levels in TG-PKR1 hearts compared to WT hearts, and hierarchical clustering separated the two groups (Fig. 1A). Functional annotation analysis of this cluster using GoMiner showed a highly significant enrichment (FDR = 0%) of lipid-associated processes. SAM analysis identified 46 probes (corresponding to 44 unique genes) with highly significant differences in gene expression between WT and TG-PKR1 hearts (FDR = 0%).

Hierarchical clustering of the samples based on the 46 expression profiles is shown in Fig. 1B. The genes with lower expression in the TG hearts included the following PPARγ signaling pathway-related genes^10^: *PPARγ* (*Pparg*), *fatty acid binding protein 4* (*Fabp4*)*/Ap2*, *UCP-1* (*Ucp1*), *Stearoyl-CoA desaturase-1* (*Scd1*), *Perilipin* (*Plin1*), *Adipoqine/adiponectin* (*Adipoq*). These genes are active in adipogenesis and were significantly enriched within this set of expression profiles (DAVID analysis, Benjamini corrected p < 0.0005) (Supplementary Material, Figure S1).

Next, the downregulated expression of these six genes in the TG hearts was demonstrated by qPCR analyses (Fig. 1C), which confirmed impaired PPARγ signaling in TG hearts. We then examined whether PKR1 signaling is a cell type-specific regulator of PPARγ expression in TG hearts. Western blot analyses of PPARγ protein levels were performed on CFP-like cells derived from cardiac explants and cardiomyocytes isolated from adult TG-PKR1 and WT hearts (Fig. 1D). PPARγ protein levels were the same in both the TG-PKR1 and WT cardiomyocytes. However, PPARγ levels were lower in the CFP-like cells derived from TG hearts (Fig. 1E). Indeed, prokineticin-2 inhibited PPARγ levels in the isolated CFP-like cells within 12 h (Fig. 1F). Taken together, these data suggest that cardiomyocyte-PKR1 signaling may regulate a novel PPARγ-related event in CFP-like cells in a paracrine manner.

### TG-PKR1 in cardiomyocytes inhibited epicardial adipose tissue (EAT) in mice fed an HFD

**Figure 2:**
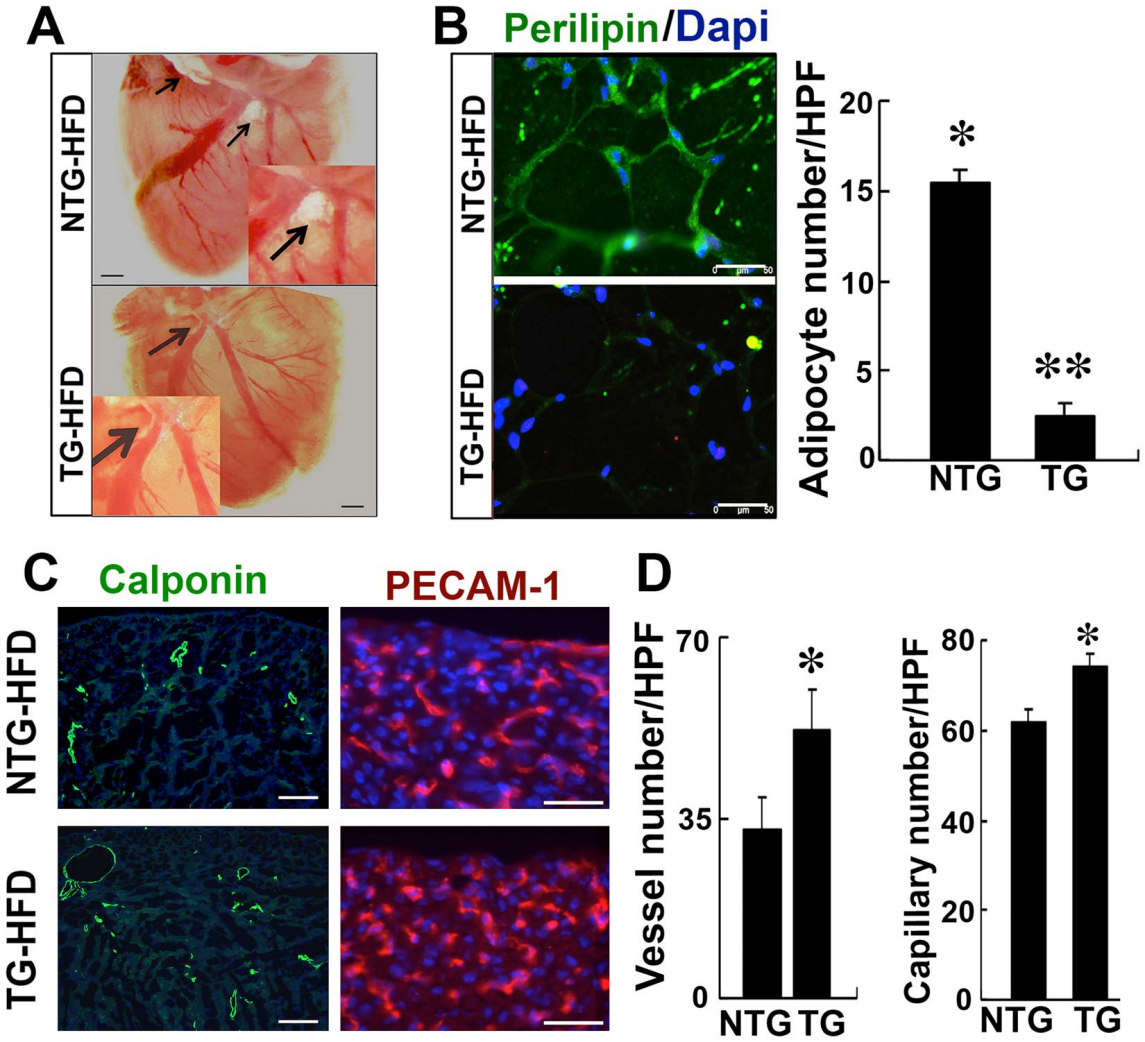
Fat tissue accumulation and the vascular network in TG-PKR1 hearts after HFD exposure. (**A**) Fat tissue deposition around the atrioventricular groove (AVG) of WT hearts and TG-PKR1 hearts after exposure to a high-fat diet (HFD) (representative of 3 hearts). (**B**) Peripilin staining of the AVG in WT and TG-PKR1 hearts. Histogram shows a few perilipin^+^ adipocytes in the TG-PKR1 hearts (10 pictures per section from 3 hearts in each group). (**C**) Smooth muscle-specific calponin and endothelial-specific PECAM-1 staining of cryosectioned heart sections. (**D**) Histogram shows that after HFD consumption, the vessel and capillary numbers are still higher in the TG-PKR1 hearts (*p < 0.05, compared to NTG-fed mice, 10 pictures per section, unpaired two-tailed Student’ s *t*-Test). All n = 5 mice/group.

To study the pathological consequences of low PPARγ levels in CFP-like cells, TG-PKR1 mice were fed an HFD. Unlike WT mice fed an HFD, TG-PKR1 mice did not develop adipose tissue around the atrioventricular grooves (AVG) (Fig. 2A). Perilipin staining of the cryosectioned hearts clearly showed that the HFD induced adipocyte development in the AVG of WT mice, whereas TG mouse hearts displayed almost no perilipin staining (Fig. 2B). Interestingly, endothelial-specific PECAM-1 and smooth muscle-specific calponin staining showed that TG hearts retained an extensive vascular network even after exposure to an HFD (Fig. 2C,D). These data indicate that cardiomyocyte PKR1 signaling may play an important role in the development of cardiac fat tissue by interacting with CFP-like cells.

### Prokineticin-2 as a secretome of cardiomyocytes overexpressing PKR1 regulates CFP-like cell fate

**Figure 3:**
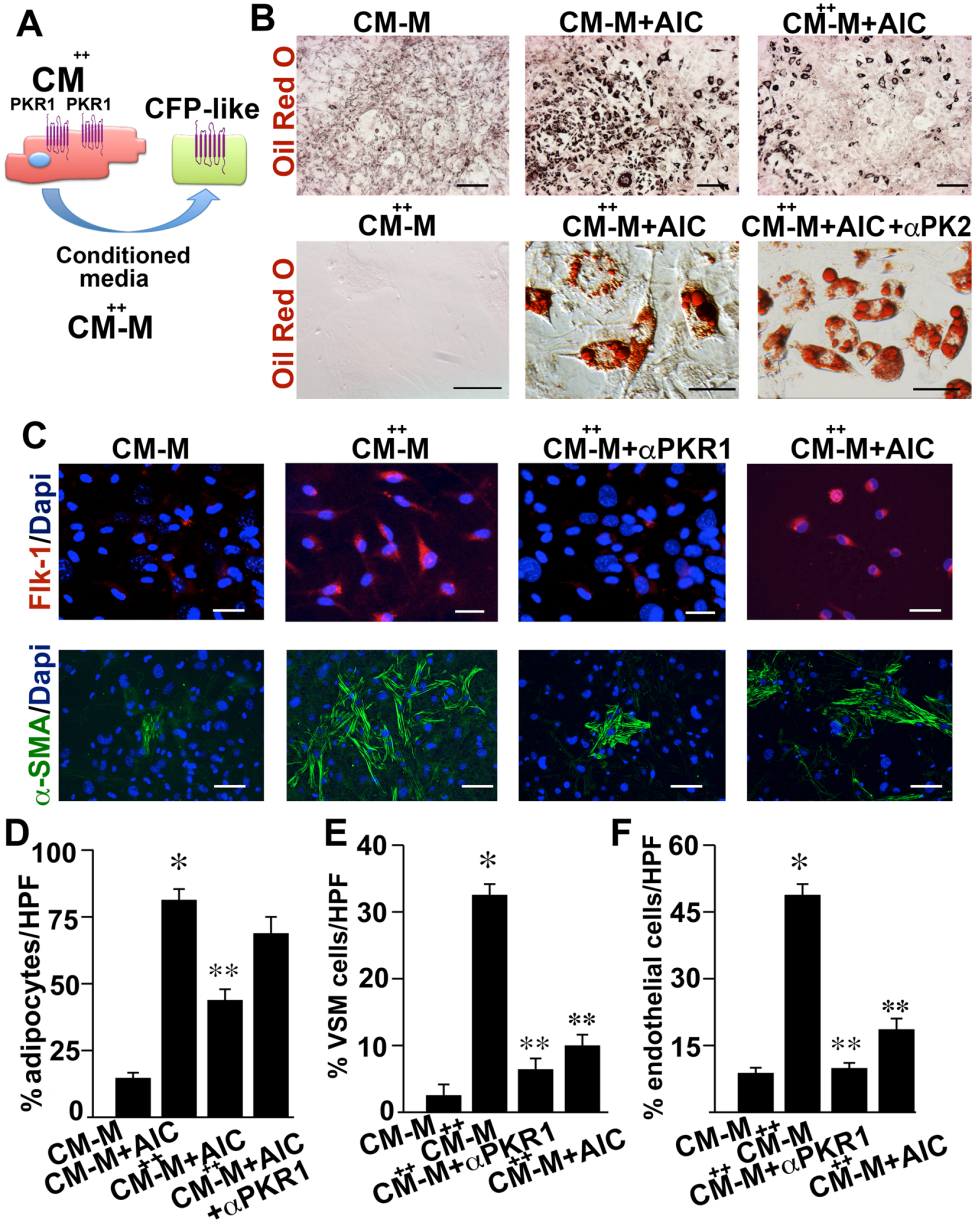
Regulation of the transformation of CF-like cells into adipocytes by a cardiomyocyte-mediated paracrine pathway. (**A**) Schematic illustration of the experimental design. Adult CF-like cells were cultured in the conditioned medium of PKR1-overexpressing cardiomyocytes (infected with adv-PKR1) (CM^++^-M) to mimic TG mouse models. Conditioned medium of cardiomyocytes (CM-M) infected with Adv-control was used as a control. (**B**) Oil Red O staining of CF-like cells cultured in CM-M alone or in CM-M supplemented with an adipogenic induction cocktail (AIC) (CM-M+ AIC), CM^++^-M alone or supplemented with AIC (CM^++^-M+ AIC) in the presence or absence of anti-PK2 antibody (α-PK2). (**C**) Endothelial-specific Flk-1 or smooth muscle actin (α-SMA) staining of CF-like cells following the treatments described above. Adipocyte (**D**), SMC (**E**) and endothelial cell (**F**) numbers are presented as histograms (*p < 0.05, compared with vehicle-treated cells; **p < 0.05, compared to AIC-treated cells; n = 6, 10 pictures per condition, unpaired two-tailed Student’ s *t*-Test). All n = 12 mice/group. The drawing in 3A was created using Servier Medical Art illustration resources (www.servier.com).

To gain insight into the cellular mechanism of the paracrine effects of cardiomyocyte-PKR1 signaling and to find a paracrine factor that is released from cardiomyocytes, we mimicked the *in vivo* TG-heart model in a cell culture system. We utilized both isolated CFP-like cells from cardiac explants (the majority of which express *Tc21* and, to a lesser extent, the *epicardial genes Wt1* and *Tbx18*) and PKR1-overexpressing cardiomyocytes utilizing adenoviruses carrying PKR1 cDNA (CM^++^) or mock cDNA (CM) (Fig. 3A). Treatment of isolated CFP-like cells with an adipocyte-induction cocktail in CM medium (CM-M) promoted adipocyte formation, which was visualized by Oil Red O staining that accumulates in lipid vacuoles (Fig. 3B central panel). When the CF-like cells were exposed to AIC-containing conditioned medium derived from CM^++^ (CM^++^-M+ AIC) cells, adipogenic conversion was abolished (Fig. 3B right panel). However, this anti-adipogenic effect was reversed upon pretreatment of cells with prokineticin-2-neutralizing antibody (α-PK2) (α-PK2+ CM^++^-M+ AIC) (Fig. 3B lower panel and 3D histogram). Conversely, exposure to CM^++^-M alone promoted the differentiation of CFP-like cells into α-SMA^+^ vascular smooth muscle cells (Fig. 3C, E histogram) and PECAM-1^+^ endothelial cells (Fig. 3C, F histogram), thus mimicking our *in vivo* TG-PKR1 model^9^. The α-PKR1 pretreatment diminished the CM^++^-M-induced vasculogenic differentiation of CFP-like cells. Thus, combined with our *in vivo* data, these data suggest that PK2 is a secretom of cardiomyocytes that controls the conversion of CFP-like cells to adipocytes and vasculogenic cells in a paracrine manner.

### PKR1 controls cell-autonomously adipogenic and vasculogenic transdifferentiation in adult tcf21^+^ CFPs

**Figure 4:**
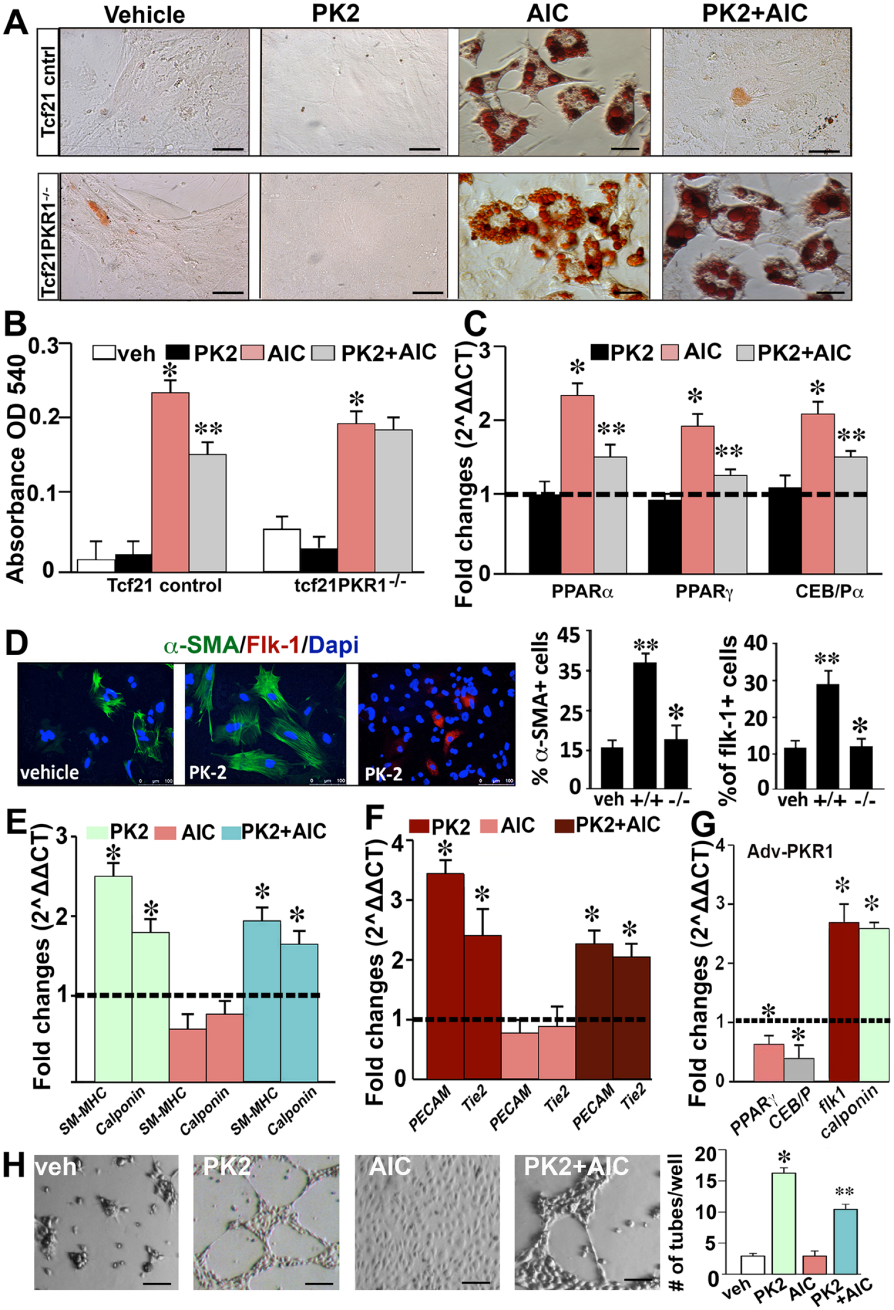
Role of Prokineticin-2 on the differentiation of tcf21^+^ CPFs. (**A**) Oil-Red-O-stained control and PKR1-deficient tcf21/Tomato^+^ FACS-isolated CPFs treated with vehicle or an adipogenic induction cocktail (AIC) (mixture of isobutanol, IBMX, and insulin) in the presence or absence of prokineticin-2 (PK2, 10 nmol/L). (**B**) Quantification of Oil-Red-O-stained lipids by spectrometric analyses in tcf21^+^ CPFs derived from wild-type (left) and PKR1^*tcf−/−*^ mice (right) (*p < 0.05, compared to vehicle-treated cells; **p < 0.05, compared to AIC-treated cells; n = 5, *two way ANOVA*). (**C**) qPCR analyses of adipogenic gene expression (*PPARα*, *PPARγ*, *and CEB/Pα*) in tcf21^+^ wild type CPFs (*p < 0.05, compared to vehicle-treated cells; **p < 0.05, compared to AIC-treated cells, n = 4). (**D**) PK2-induced vasculogenic differentiation of tcf21^+^ CPFs as evaluated by endothelial-specific Flk-1 and smooth muscle cell-specific α-SMA co-staining. The histograms show the quantitative analyses of vasculogenic cell numbers (**p < 0.05, compared to vehicle-treated wild-type cells; *p < 0.05, compared to PK2-treated wild-type cells; n = 6, 10 pictures per condition). (**E**) Quantitative real-time PCR (qPCR) analyses of smooth muscle-specific (*calponin* and *SM-MHC*) and (**F**) endothelial cell-specific gene expression levels (*Tie2* and *Pecam-1*) upon PK2, AIC and AIC+PK2 treatment (*p < 0.05, compared to vehicle-treated cells, n = 4). (**G**) qPCR analyses of vasculogenic (*Flk-1 and calponin*) and adipogenic gene expression levels (*PPARα* and *CEB/Pα*) in tcf21^+^ CPFs infected with adenoviruses carrying PKR1 cDNA or control cDNA (*p < 0.05, compared to Adv-control cells; n = 5). (**H**) Vessel-like formation on Matrigel after plating cultured tcf21^+^ CPFs treated with PK2 (10 nmol/L), AIC, AIC+PK2 or vehicle. In the histogram, *p < 0.05, compared to vehicle-treated cells; and **p < 0.05, compared to PK2-treated cells; n = 5. All n = 12 mice/group. All error bars represent the s.e.m, unpaired two-tailed Student’ s *t*-Test was applied. Dash lines show expression of genes in the only vehicle treated cells.

To further establish the adipogenic and vasculogenic potential of CFPs and the role of prokineticin-2 during these events, we used a pulse-labeled genetic lineage-tracing strategy, in which tcf21 drives CreERT2 expression in the majority of CFPs in adult mice. In the presence of the inducing agent tamoxifen, CreERT2 indelibly activates the Rosa26Tomato (TM) reporter to label CFPs with red fluorescent protein. We then isolated Tomato-expressing tcf21^+^ CFPs by FACS and examined the role of prokineticin-2 on the adipocyte and vascular cell formation capacity of these cells upon adipogenic induction. Tcf21^+^ CFPs differentiated into adipocytes upon AIC treatment (Fig. 4A). Quantification of Oil-Red-O-stained lipids confirmed the increase in lipid formation within seven days of adipogenic differentiation (Fig. 4B). Mouse tcf21^+^ CFPs showed no sign of adipocyte differentiation in the absence of an AIC. Pretreatment of tcf21^+^ CFPs with prokineticin-2 inhibited AIC-induced adipocyte differentiation (Fig. 4B left histogram). This effect of prokineticin-2 was completely absent in the PKR1 deficient (PKR1^*tcf*−*/*−^) CFPs indicating the involvement of PKR1 (Fig. 4A,B right histogram). It should be noted that AIC-mediated adipogenesis was slightly reduced in the PKR1 deficient CFPs as compare to wild type cells. Accordingly, prokineticin-2 pretreatment inhibited the expression of *PPARα*, *PPARγ*, and *CEB/Pα*, which was elevated in tcf21^+^ wild type CFPs cultured in media containing AICs alone (Fig. 4C).

In parallel experiments, we assessed the potential of tcf21^+^ CFPs to differentiate into functional vasculogenic cells *in vitro* (Fig. 4D, histograms). Quantification of Flk-1^+^ endothelial^11^ and α-SMA^+^ vascular smooth muscle cells^12^ showed that prokineticin-2 alone induces vasculogenic differentiation in tcf21^+^ CPFs after seven days compared to vehicle- or AIC-treated tcf21^+^ CPFs. Prokineticin-2 treatment also resulted in higher expression of vascular-specific genes, such as smooth muscle myosin heavy chain (SM-MHC), calponin, PECAM-1 and Tie2, compared to treatment with vehicle or AICs (Fig. 4E, F).

In cultured PKR1-overexpressing tcf21^+^ CPFs caused by Adv-PKR1 infection, the expression of *PPARγ* and *CEB/Pα* was decreased, whereas *Flk-1* and *calponin* expression was increased (Fig. 4G), indicating the cell-autonomous regulation of tcf21^+^CF cell fate by PKR1. Indeed, endothelial cells from prokineticin-2-induced tcf21^+^ CF differentiation were able to form tube-like structures on Matrigel (Fig. 4H and histogram), demonstrating that these differentiated cells also possess functional characteristics of endothelial cells. However, prokineticin-2 treatment reduced the expression of myofibroblast markers, such as fibronectin and collagen-1, in these cells (Supplementary Material, Figure S2). In the PKR1-deficient cells, the expression of myoblast genes (*e*.*g*., fibronectin and collagen) was not altered by prokineticin-2 treatment (Supplementary Material, Figure S2).

These results indicate that tcf21^+^ CPFs can be the source of adipocytes and vasculogenic cells, which can be controlled by prokineticin/PKR1 signaling.

### Genetic PKR1 inhibition in tcf21^+^ CPFs promotes excessive adipose tissue development in mice fed an HFD

**Figure 5:**
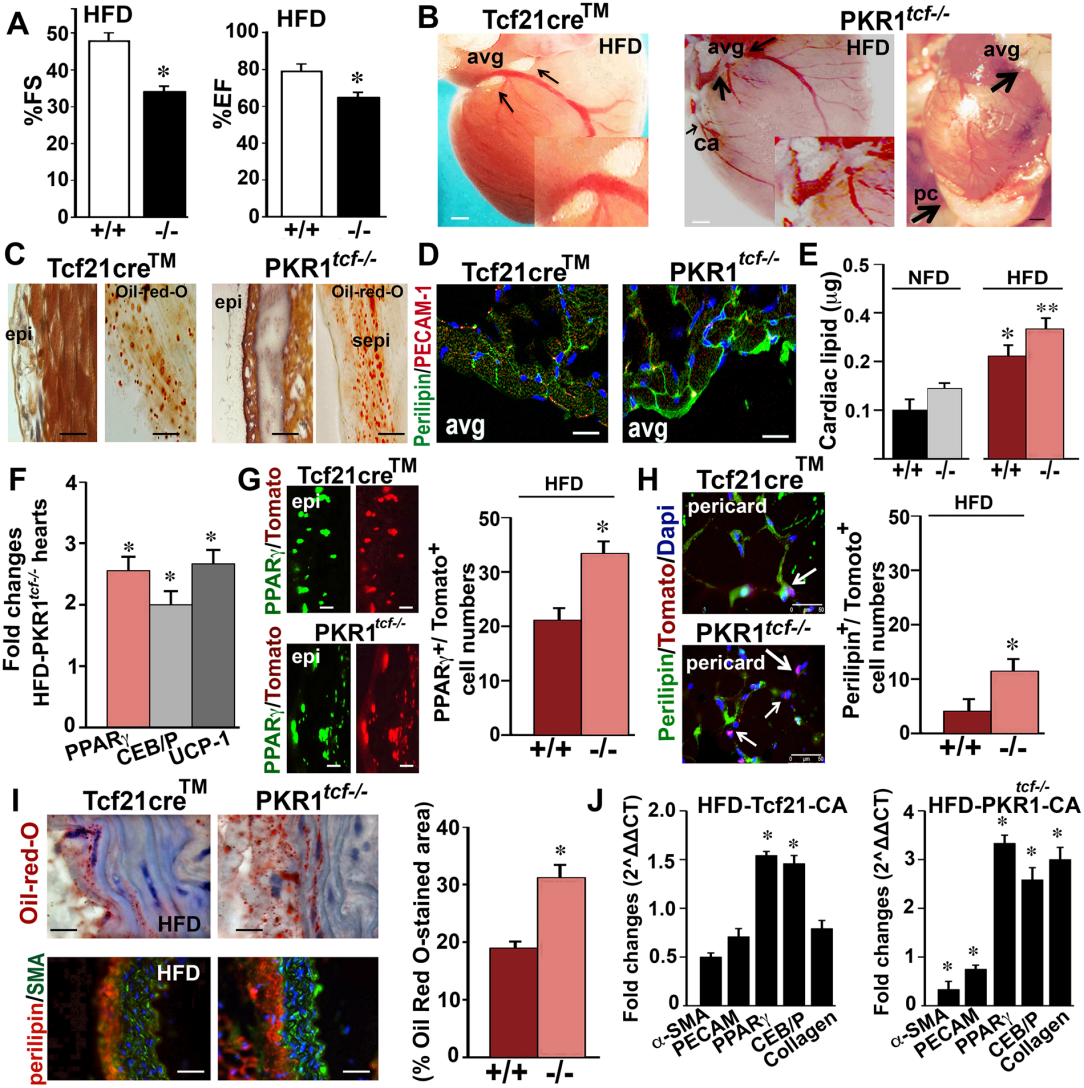
Fat tissue development in PKR1^*tcf*−*/*−^ hearts after HFD exposure. (**A**) Ejection Fraction (EF) and fractional shortening (FS) of the HFD-fed mice (*p < 0.05, compared to HFD-fed control mice, n = 6–8; *two way ANOVA*). (**B**) Localization of adipose tissue in the atrioventricular groove (avg) of HFD-fed control (tcf21cre) mice and in the avg as well as around the coronary artery (ca) and the pericardium (pc) of PKR1^*tcf−/−*^ mice (*n* = *6 mice/group*). (**C**) Malory tetrachrome (left) and Oil Red O (right) staining of cardiac sections derived from control and PKR1^*tcf−/−*^ mice (n = 6 mice/group) after HFD exposure; subepicardium (sepi). (**D**) Perilipin and PECAM-1 staining of fat tissue around the avg in mice of both genotypes (n = 4, each) that were fed an HFD. (**E**) Histogram shows extracted cardiac lipid levels in the hearts (*p < 0.05, compared to control; **p < 0.05, compared to HFD-fed control mice, *n* = 6 hearts/group; *two way ANOVA*). (**F**) qPCR analyses for adipose tissue markers (*PPARγ*, *CEB/Pα* and *UCP-1*) in HFD-fed mouse hearts. *p < 0.05, compared to control mice (*n* = *6 mice/group*, unpaired two-tailed Student’s *t*-test). (**G**) Co-immunostaining of PPARγ and Tomato in ventricular sections from control and PKR1^*tcf−/−*^ mice after HFD exposure. Histogram shows number of PPARγ^+^ cells in the heart. *p < 0.05, compared to control mice (10 pictures for each section, 6 mice/group, unpaired two-tailed Student’s *t*-test). (**H**) Perilipin-A staining of pericardial adipose tissues in mice of both genotypes that were fed an HFD (*10 pictures for each heart section*, 6 mice/group). Some of the perilipin^+^ cells are also Tomato^+^. (**I**) Oil Red O- (upper) or perilipin-A (lower panel)-stained perivascular adipose tissue (pvat) in the HFD-fed PKR1^*tcf−/−*^ and control hearts, (*p < 0.05, compared to control mice, 10 pictures for each heart section, n = 6 mice/group, unpaired two-tailed Student’s *t*-test). (**J**) qPCR analyses of RNA extracted from the coronary arteries of NFD- (left) and HFD (right)-fed mice for adipose tissue markers (*PPARγ*, *CEB/Pα* and *UCP-1*) and vasculogenic cell markers (PECAM-1 and *α*-SMA) as well as fibroblast markers (collagen). *p < 0.05, compared to control mice (n = 6 mice/group, unpaired two-tailed Student’s *t*-test).

To gain insight into the role of PKR1 in CPFs *in vivo*, we generated PKR1^*tcf*−*/*−^ mice (Supplementary Material, Figure S3A) by crossing a tcf21^ERT^cre^™^ driver mouse line with the PKR1 floxed mouse line and explored the pathophysiological consequences of PKR1 deficiency on the development of adipose tissue and vascularization upon the administration of an HFD. Epicardial tracing was performed in 8-week-old adult tcf21Cre^ER^xRosa26^TM/+^ (control) and PKR1^*tcf*−*/*−^ mice following TMX injection for 3 consecutive weeks. The ablation of PKR1 was confirmed by PKR1 immunostaining in isolated tcf21^+^ cells (Supplementary Material, Figure S3B). Then, mice were treated with an NFD or HFD for 4 weeks. Body weight and abdominal white adipose tissue (WAT) weight of mice treated with an HFD were equally increased within 4 weeks in both control and PKR1^*tcf*−*/*−^ mice (Supplementary Material, Figure S3C). Impaired systolic (fractional shortening, %FS) and diastolic (ejection fraction, %EF) function in the PKR1^*tcf*−*/*−^ mice was evident only after HFD exposure (Fig. 5A, Table 1).

**Table 1:** Echocardiographic analyses of mice (n = 6–8).

Genotype	tcf21 (control)		PKR1^*tcf*−*/*−^	
	NFD	HFD	NFD	HFD
Heart rate	475 ± 15	435 ± 12	445 ± 15	460 ± 15
LVEDD (mm)	3.45 ± 0.1	3.79 ± 0.2	3.38 ± 0.1	3.88 ± 0.1^#t^*
LVESD (mm)	1.87 ± 0.3	1.90 ± 0.2	1.90 ± 0.2	2.5 ± 0.2^#t^*
Fractional shortening (%)	45.9 ± 5	49.5 ± 3	43.01 ± 3	35.83 ± 3^#t^*
Ejection fraction (%)	74.77 ± 3	79.5 ± 4	73.27 ± 3	65.71 ± 3^#t^*
IVRT (ms)	16 ± 2	18 ± 2	17 ± 2	23 ± 2^#t^*
LV mass	125 ± 9	150 ± 12^t^	135.27 ± 8	187 ± 8^#t^*

*Shows p < 0.05 compare to the HFD fed control mice (ANOVA).

^t^Shows p < 0.05 compare to the NFD fed control mice.

^#^Shows p < 0.05 compare to the NFD fed PKR1^*tcf*−*/*−^ mice.

Histological analyses of the mouse hearts revealed that the adipose tissue was significantly elevated in the AVG of tcf21cre control mice fed an HFD (Fig. 5B left). However, in the hearts of PKR1^*tcf*−*/*−^ mice, adipose tissue accumulated both around the AVG and the adventitia of the coronary artery branches after HFD exposure (Fig. 5B middle). We also observed severe pericardial fat accumulation in 60% of PKR1^*tcf*−*/*−^ mouse hearts (Fig. 5B right). Tcf21cre^TM^ lineage-derived adipose tissue was sparingly present in the AVG of control and PKR1^*tcf*−*/*−^ adult mice fed an NFD (Supplementary Material, Figure S3D). These findings indicate the presence of minimal fibroblast-adipocyte-transformation in the hearts of both adult control and mutant mice.

Malory tetrachrome-stained heart sections showed severe fat tissue accumulation in the subepicardium of the PKR1^*tcf*−*/*−^ hearts (Fig. 5C left panel). Oil Red O staining confirmed the presence of lipid accumulation around the subepicardial area in PKR1^*tcf*−*/*−^ hearts after HFD exposure (Fig. 5C right panel). Perilipin-A-stained heart sections revealed increased lipid accumulation in the AVG of PKR1^*tcf*−*/*−^ hearts after HFD exposure (Fig. 5D). Accordingly, the extracted cardiac lipid levels were also higher in the hearts of PKR1^*tcf*−*/*−^ mice fed an HFD (Fig. 5E). In concert with these findings, the expression of adipogenic genes (*PPARγ*, *CEB/Pα* and *UCP-1*) was increased by 2-fold in the hearts of HFD-fed PKR1^*tcf*−*/*−^ mice compared to that of HFD-fed tcf21cre^TM^ control mice (Fig. 5F). Tcf21Cre^TM^-mediated recombination was highly efficient, and we observed a virtually complete labeling of PPARγ-positive adipose tissue using this lineage marker (Tomato^+^/tcf21^+^) following HFD treatment (Fig. 5G). The hearts of HFD-fed PKR1^*tcf*−*/−*^ mice displayed a 15 ± 3% increase in the number of Tomato^+^/PPARγ^+^ cells compared to control hearts (Fig. 5G histogram). Perilipin^+^ staining of the pericardial fat tissues in the PKR1^*tcf*−*/*−^ hearts (Fig. 5H) revealed that 13 ± 2% of the pericardial fat tissue was also positive for the Tomato^+^ tcf21 lineage marker. However, only 5 ± 1% of the pericardial fat tissue was positive for the Tomato^+^ tcf21 lineage marker in controls. Altogether these data indicate that the tcf21^+^ CPFs are also a source of adipocytes in EAT, perivascular adipose tissue (PVAT) and, to a lesser extent, pericardial fat tissue.

Oil Red O staining (Fig. 5I upper, and histogram) and perilipin immunostaining (Fig. 1J lower) also revealed severe fat accumulation around the perivascular tissue in the hearts of HFD-fed PKR1^*tcf*−*/*−^ mice compared to that of HFD-fed tcf21cre control mice. Perilipin^+^ stained cell numbers in the PKR1^*tcf*−*/*−^ pericardial fat tissues were four times higher (75 ± 5%) than that of tcf21cre controls (15 ± 8%) after HFD exposure (Fig. 5I). The coronary arteries of HFD-fed PKR1^*tcf*−*/*−^ mice had higher levels of adipogenic gene expression (*PPARγ*, *CEB/Pα* and *UCP-1*), whereas the expression of vasculogenic genes (*Flk-1 and calponin*) was lower (Fig. 5J).

These data indicate that a lack of PKR1 signaling in tcf21^+^ CPFs induces excessive fat accumulation in EAT, PVAT and pericardial fat tissue and may affect the development of the vascular network.

### PKR1^*tcf*−*/*−^ mice displayed impaired vascularization after HFD exposure

**Figure 6:**
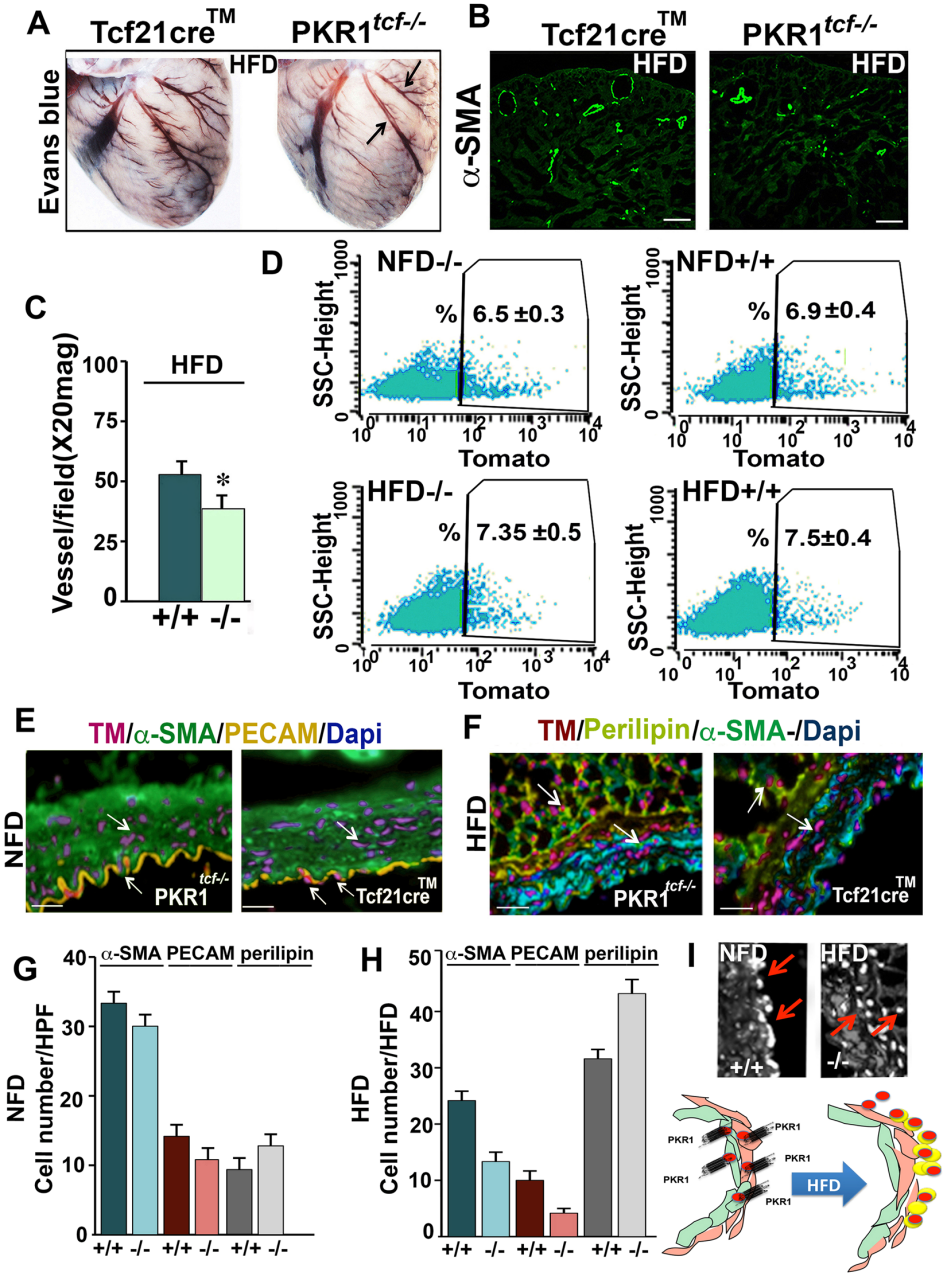
Vessel development in HFD-treated PKR1^*tcf*−*/*−^ mice. Evans blue staining of the hearts from HFD-fed PKR1^*tcf*−/−^ and control mice (*n* = 4). (**B**) α-SMA staining of vessels on cryosectioned heart tissue. (**C**) Histogram shows the vessel numbers in the cryosectioned hearts of control and PKR1^*tcf*−/−^ mice after HFD exposure (*p < 0.05, compared to control mice, 10 pictures per section were analyzed, n = 6 mice/group, *t-test*). (**D**) FACS-isolated Tomato cells in NFD- and HFD-fed control (/+) or mutant (−/−) mouse hearts (p > 0.05, no difference between groups, n = 6 mice/group, unpaired two-tailed Student’s *t*-test). (**E**) Staining of coronary arteries from NFD-fed mice with α-SMA, PECAM-1, and perilipin antibodies. (**F**) Staining of coronary arteries from HFD-fed mice with α-SMA, PECAM-1, and perilipin. Histogram shows α-SMA^+^/Tomato^+^, PECAM-1^+^/Tomato^+^, and perilipin^+^/Tomato^+^ cell numbers in NFD- (**G**) and HFD-exposed (**H**) control (+/+) and mutant mouse (−/−) hearts. (*p < 0.05, compared to control mice, 10 sections per section were analyzed, n = 6 mice/group, unpaired two-tailed Student’s *t*-test). (**I**) Tomato^+^ cell localization in the coronary arteries of control and mutant mice exposed to an NFD or HFD. Schematic illustration of the tcf21^+^ CPFs from control mice exposed to an NFD (left) and the tcf21^+^ CFPs from PKR1^*tcf*−*/*−^ mice exposed to an HFD (right). The drawing in 6I was created using Servier Medical Art illustration resources (www.servier.com).

Next, we investigated whether the vascular network of PKR1^*tcf*−*/*−^ hearts was affected. Indeed, NFD-fed PKR1^*tcf*−*/*−^ mice did not exhibit significant alterations in vascular density at 12 weeks of age (Supplementary Material, Figure 4). However, after HFD exposure, the hearts of PKR1^*tcf*−*/*−^ mice exhibited less coronary vascularization as detected by Evans blue (Fig. 6A) and α-SMA (Fig. 6B) staining (Fig. 6C histogram).

We then determined the number of tcf21^+^ CPFs in these hearts. FACS analyses revealed that the number of tcf21^+^ CPFs in PKR1^*tcf*−*/*−^ or control tcf21cre^TM^ mice after HFD exposure was not significantly altered (Fig. 6D). However, the localization of Tomato^+^ cells was altered by HFD exposure. In the coronary arteries of control mice, some of the PECAM^+^ endothelial cells and α-SMA^+^ vascular smooth muscle cells were also Tomato^+^ (Fig. 6E). These ratios were slightly reduced in the PKR1^*tcf*−*/*−^ coronary arteries (Fig. 6E,G). However, Tomato^+^ cells were predominantly accumulated in the perilipin^+^ adipose tissues of control coronary arteries after HFD treatment (Fig. 6F left panel). The shift in the location of Tomato^+^ cells was more profound in the coronary arteries of PKR1^*tcf*−*/*−^ mice (Fig. 6F,H). A robust increase in the perilipin^+^/Tomato^+^ cell numbers was observed in the PKR1^*tcf*−*/*−^ hearts after HFD exposure (Fig. 6I).

Altogether, these data indicate that HFD exposure, together with impaired PKR1 signaling promote the conversion of tcf21^+^ CPFs into fibroblast-fat tissue (Fig. 6I). This was demonstrated in PKR1^*tcf*−*/*−^ mice by excessive fat tissue development at the AVG and around the perivascular area of the coronary arteries as well as the low level of vascularization.

## Discussion

**Figure 7:**
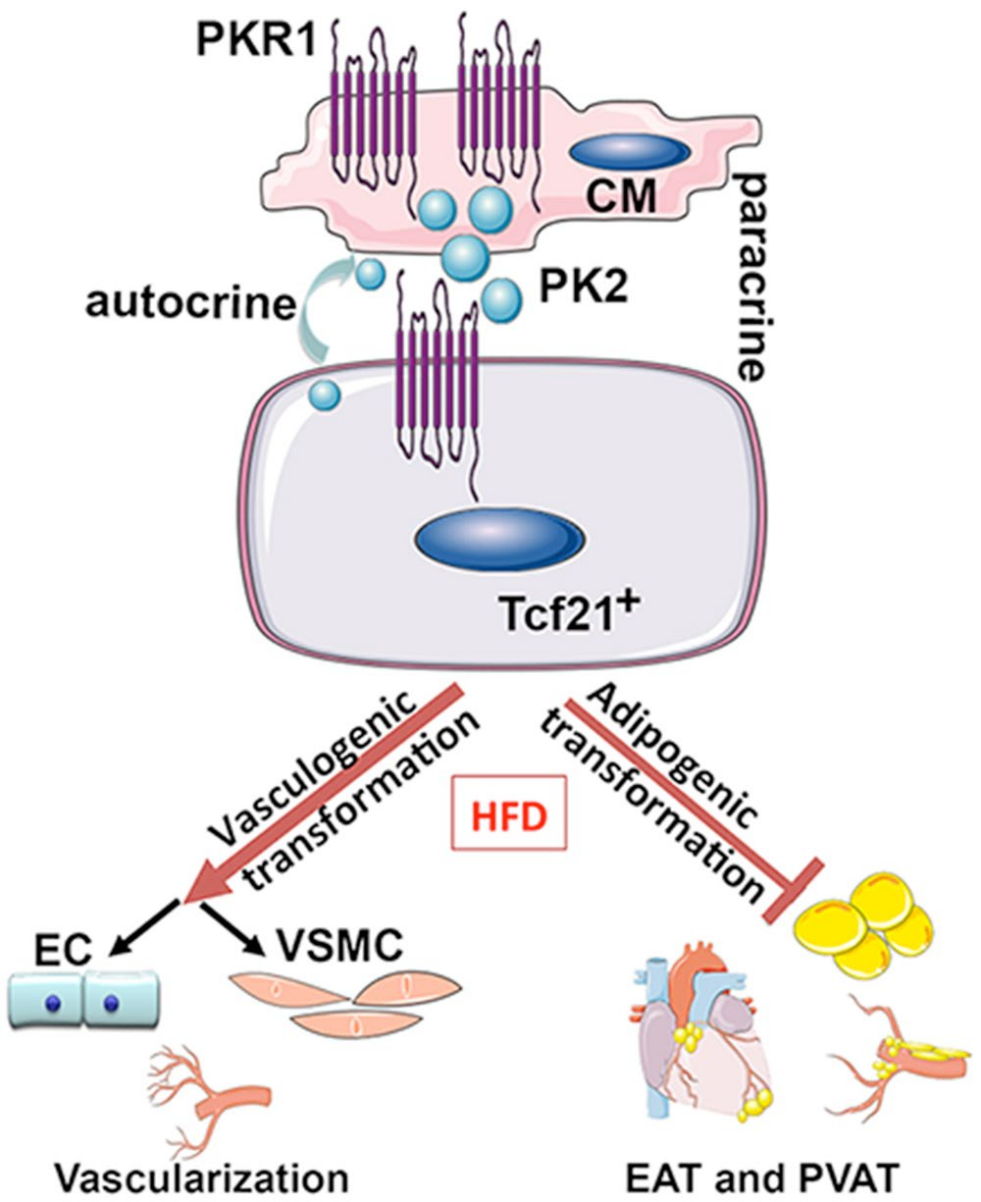
Prokineticin/PKR1 signaling drives tcf21^+^ cell fate. Schematic illustration showing that PKR1 signaling in tcf21^+^ CPFs suppresses fibroblast-adipocytes-transformation and promotes fibroblast-vasculogenic-transformation via autocrine and paracrine pathways through prokineticin-2 following consumption of a high-fat diet (HFD). The drawings were created using Servier Medical Art illustration resources (www.servier.com).

The capacity of CPFs to differentiate into smooth muscle cells or myofibroblasts is well established, but their vasculogenic potential has remained controversial. The adipogenic potential of CPFs has not been previously described. Our study revealed that cardiac fat (EAT and PVAT) and the vascular network originate from a common tcf21^+^ lineage and are controlled in an autocrine and paracrine manner by PKR1. Here, we identified a novel paracrine regulatory mechanism of CPFs whereby cardiomyocytes secretome prokineticin-2 controls the conversation of CPFs to adipocytes. We showed that prokineticin-2/PKR1 signaling enables the reprogramming of tcf21^+^ CPFs to adopt vascular-cell-like characteristics and to suppress cardiac fat accumulation under high-calorie diet conditions. We also showed that vasculogenic and adipogenic imbalances due to lack of PKR1 disturbs cardiac function *in vivo* (Fig. 7), a phenomenon attributed to an insufficient supply of oxygen under consumption of high calories.

We showed that the prokineticin-2, as a secretome of myocardium is a potent suppressor of CFP-fat-transformation. The other cardiomyocyte secretome such as Atrial Natriuretic peptide has been shown to promote adipogenesis in epicardium^5^. Signals from cardiomyocytes can regulate EMT and the subsequent differentiation of EPDCs^13^. Paracrine factors produced by cardiomyocytes, such as folistatin-like 3 (fls-3), have been shown to contribute to the adhesion and proliferation of CPFs as well as collagen production in CPFs^14^. Placental growth factor (PGF) secreted from cardiomyocytes affects non-cardiomyocytes (mainly cardiac fibroblasts) and promotes cardiac adaptive responses after pressure overload^15^.

Here, we identified a novel cardiomyocyte secretome, prokineticin-2, controlled by PKR1 signaling controls the adipogenic conversion of CF-like non-cardiomyocytes in adult hearts. Low PPARγ signaling in the CF-like cells of TG-PKR1 mice leads to the lack of EAT development under HFD exposure, as previously observed in the PPARγ-knockout mice^16^. PPARγ is the principal inducer of adipogenesis and a marker of mature adipocytes^17^. This function is consistent with the adipogenic suppressor activity of prokineticin-2/PKR1 signaling in pre-adipocytes and mesenchymal stem cells^7^. Prokineticin-2 binds to PKR1 in CPFs and suppresses transcriptional adipogenic factors *C/EBPα*, *PPARγ and UCP-1*, and promotes to the trans differentiation of CPFs into vasculogenic cell types.

Previously, Wt1^+^ cells^18^ and Tbx18^+^ cells^19^ have been shown to promote the development of EAT in adult hearts after myocardial infarction. Insulin-like growth factor 1 receptor (IGF1R) activation activates EAT development, and IGF1R inhibition reduces Wt1^+^ cell differentiation into adipocytes after myocardial infarction^20^. Here, we showed that tcf21^+^ CFPs originating from epicardium contribute to adipogenesis in adult heart under HFD exposure, since the recombinase being induced at the adult stage and labeled by the tomato in PPARγ^+^ adipocytes in our experiments. The observation of tcf21^+^ cells in both subepicardial and perivascular adipose tissue suggests that both fat depots have a common cellular origin. Tcf21 positive EAT can migrate and participate in the formation of pericardial adipose tissue, as 5–13% of pericardial adipose tissue was labeled with tcf21^TM^cre. However, we cannot fully exclude the possibility that the tcf21positive cells lining the chest cavity can differentiate into adipocytes and infiltrate the pericardial fat tissue. EAT, PVAT and, to a lesser extent, pericardial adipose tissue are novel types of adipose tissues that are mainly derived from tcf21 positive CPFs and play an important role in the pathogenesis of cardiovascular diseases. However, the mechanisms regulating cardiac adipose tissue expansion might be distinct; for instance, high calorie consumption is required in the tcf21^+^ cells lacking PKR1 signaling. Cardiac fat tissue expansion could be caused by nutrient excess in which epicardium and pericardium becomes inflated with lipids resulting from the subcutanious adipose tissue. This possibility is discarded by our observation that PKR1^−/−^ mice, with intermittent HFD exposure indeed developed EAT and PVAT at the expense of significant subcutenous AT formation. However, PKR1 intake mice in the context of HFD developed fat accumulation in the atrial ventricular groove and subepicardium. Massive adipose infiltration of the posterior wall of left atria has been described in sheep with moderate obesity (57). Despite the number of tcf21^+^ cells were not altered by lack of PKR1 signaling in tcf21^+^ CPFs, and by a HFD exposure alone, the adipocyte conversion of tcf21^+^ CPFs was higher than vascular cell differentiation in the HFD fed PKR1^−/−^ hearts.

Wt1^+^ and Tbx18^+^ epicardial cells are also sources of CPFs, undifferentiated subepicardial mesenchymal cells, coronary endothelium and coronary VSMCs^21^. Previously, angiogenic factors, such as vascular endothelial growth factor A (VEGFA)^22^ and thymosin B4 (TB4)^23^, have been shown to induce the differentiation of Wt1^+^ cells into endothelial and/or vascular smooth muscle cells and fibroblasts. Notably, previous studies have shown that PKR1 is necessary for the proliferation of the Wt1^+^ lineage and for their subsequent differentiation into endothelial cells and vascular smooth muscle cells during heart development^24^. Consistent with these findings, we showed that the activation or overexpression of PKR1 cell autonomously increases the differentiation of adult tcf21^+^ CPFs into endothelial cells and vascular smooth muscle cells by regulating the expression of vascular-specific genes (*PECAM-1*, *Flk-1*, *calponin and SM-MHC*). Indeed, the tcf21^+^ cells may also represent precursor of vasculogenic cells and adipocytes, since tcf21 is expressed not only in fibroblast progenitors, but also in various vasculogenic and adipocyte precursors detected by both tcf21 and cell specific markers, in tcf21cre mice hearts under HFD exposure as well as TGPKR1 mice hearts^9^. Thus, the isolated tcf21-positive cells may represent a mixed pool of cells, including mesencymal progenitors and precursors with angiogenic potential, that upon prokineticin2/PKR-1 signaling could differentiate into several different cell types. This phenomenon has been shown for MSCs. Although the MSC lines lacked endothelial potential, endothelial cells were derived from mesenchymomagioblasts precursors^25^. Altogether, these data suggest that PKR1 promotes vasculogenic differentiation via the tcf21^+^ lineage in the adult heart. The mechanism in which PKR1 signaling regulates adipogenic and vasculogenic differentiation of CPF progenitor/precursors is currently under investigation in our laboratory.

What are the mechanisms behind the cardiac fat deposition and coronary vascularization defects that lead to cardiac dysfunction? It has been found that the cardiac fat deposition varies among patients and could depend on clinical conditions such as aging, left ventricular dysfunction, or atrial fibrillation^26^. This variation is in agreement with the current idea that adipose depots are a common component of the myocardium that could regulate the metabolic or oxidative status of neighboring myocardium. Hence, the imbalance between adipogenic and vasculogenic transformation under the HFD exposure could become deleterious for cardiomyocytes, leading to cardiac dysfunction as observed in PKR1^*tcf*−*/*−^ hearts.

The anti-contractile effect of high-calorie intake was abrogated by the development of excessive fat tissue and an ischemic environment due to the loss of capillary and vessel networks in PKR1^*tcf*−*/*−^ hearts. These changes are subject to the maladaptive adipocyte biology of obesity^27^. Disruption of the balance between adipogenic and vasculogenic differentiation pathways in tcf21^+^ CPFs is a possible mechanism underlying PKR1 deficiency-mediated cardiac dysfunction. However, although PKR1^*tcf*−*/*−^ mice exhibited a slight decrease in vascularization, they exhibit detectable cardiac dysfunction, indicating that impaired vascularization itself is not sufficient to promote cardiac dysfunction in 12-week-old mice. Likewise, tcf21cre^TM^ control mice fed an HFD did not show any significant alterations in cardiac function, indicating that cardiac fat accumulation alone cannot impair cardiac function in our mouse models. Altogether, these data clearly showed that impaired ventricular function in PKR1^*tcf*−*/*−^ mice is due to an increase in epicardial and perivascular adipose tissue and insufficient vascular perfusion following HFD exposure.

In summary, we show that tcf21^+^ CF-to-fat transition contributes to EAT, PVAT development under HFD consummation. Tcf21^+^ CF-to-vasculogenic transition is important to maintain adequate perfusion of heart. We provide evidence that the switch between CFP-to-fat transition and CFP-to–vasculogenic transition is controlled by prokineticin-2 secreted by the myocardium as well. Previous studies have shown that prokineticin-2 or PKR1 levels are altered in human patients with abdominal aortic rupture^28^ during end‐stage cardiac failure^29^ and in adipose tissues from obese human patients^7^. Prokineticin-2 reduces food intake and body weight in a mouse model of human obesity^30,31^, PKR1 agonist improves heart function after MI^32^, and PKR1 signaling redirects Wt1^+^ epicardial cells fate. Thus, the targeting of PKR1 represents a novel approach to treat treating ischemic heart diseases and obesity.

## Methods

The methods were carried out in accordance with the approved guidelines. All animal work experiments were was performed under using protocols approved by the Direction des Services Vétérinaires du Bas-Rhin, France (Authorization N° B67-274) and the French (APAFIS#4708) and European regulation-approved protocols from Directive 2010/63/EU of the European Parliament on the protection of animals used for scientific purposes (EU0064).

### Generation of genetically manipulated mice

Generation of TG-PKR1^33^ and PKR1^fl/fl^ ^34^ mice has been previously described. *Tcf21* ^*iCre/*+^ mice^35^ were kindly provided by Eric N. Olson (University of Texas Southwestern Medical Center, Dallas, TX, USA). The *R26R* ^*Tomato ™*^ reporter mouse strain (Jackson laboratory) was used in the present study. PKR1^fl/fl^ mice were bred with *tcf21* ^*iCre/*+*TM*^ mice to create PKR1^*tcf*−*/*−^ mice. Mice were maintained on a C57BL6/J background, and data for each experiment were deduced collected from a minimum of three nulls null mice and three littermate controls. Tamoxifen (TMX) was dissolved in ethanol and then emulsified it in sesame oil with by sonication. A 200- μl volume of freshly emulsified TMX (0.12 mg/g body weight) was administered to adult *tcf21* ^*iCre/*+*Tomato*^ mice once per weekly for 3 weeks to induce CreERT2-mediated recombination. After the induction of *Cre* recombinant induction with TMX, the mice were fed a high-fat diet (HFD, 60% fat, Scientific Animal Food & Engineering, SAFE, U8954P Version 0130) or a normal-fat diet (NFD, 4,5% fat, chow diet, SAFE) for 4 weeks. The primers used for genotyping and genotyping analyses are shown in Table 2. Mice (n = 6 per group) were euthanized by cervical dislocation, and tissues were collected.

**Table 2:** Primers used for real-time PCR and genotyping.

Gene name	Forward	Reverse
Tcf21iCre	GCTTCCGATATCCAGATCCAGAC	CAAACCCTAGCACAAATCACTCGC
PKR1fl/fl	GACTGGACATCTAGTGGTAGTCAGG	GGGTGTGAGGTGGGATTAAGTCAC
β actin	CATCTTGGCCTCACTGTCCA	GGGCCGGACTCATCGTACT
GAPDH	AACGACCCCTTCATTGAC	TCCACGACATACTCAGCAC
C/EBPβ	CAAGCTGAGCGACGAGTACA	CAGCTGCTCCACCTTCTTCT
Fabp4/aP2	ACA CCG AGA TTT CCT TCA AAC TG	CCA TCT AGG GTT ATG ATG CTC TTC A
Scd-1	TCTGGGAGAGTGCTGACAAAAA	CTGCTGAGGATCCCCAAATACT
Adiponectin	GCACTGGCAAGTTCTACTGCAA	GTAGGTGAAGAGAACGGCCTTGT
PPARγ	GTGCCAGTTTCGATCCGTAGA	GGCCAGCATCGTGTAGATGA
Perilipin	GGCCTGGACGACAAAACC	CAGGATGGGCTCCATGAC
Ucp-1	ACT GCC ACA CCT CCA GTC ATT	CTT TGC CTC ACT CAG GAT TGG
Tie-2	ATGTGGAAGTCGAGAGGCGAT	CGAATAGCCATCCACTATTGTCC
Flk-1	GCCCTGCTGTGGTCTCACTAC	CAAAGCATTGCCCATTCGAT
Pecam-1	GAGCCCAATCACGTTTCAGTTT	TCCTTCCTGCTTCTTGCTAGCT
SM-MHC	GGTCGTGGAGTTGGTGGAAA	CTGCCATGTCCTTCCACCTTAG
Calponin	AGGCCAACGACCTGTTTGAA	CACATTGACTTTGTTTCCTTTTGTCT
Fibronectin	ATGTGGACCCCTCCTGATAGT	GCCCAGTGATTTCAGCAAAGG
Col1a1	GCCAAGAAGACATCCCTGAAG	TGTGGCAGATACAGATCAAGC

### Gene expression profiling

RNA from a total of six animals (3xWTn = 3 wild-type (WT); n = 3 3xTG-PKR1) was hybridized to Agilent Mouse Gene Expression Microarrays. Fluorescence values corresponding to raw expression data were extracted. The values for the positive- and negative negative-control probes were removed. Non-linear effects, such as background or saturation, were corrected by with the Locally-Weighted regression Scatterplot Smoother (LOWESS) method^36^ against a median profile of all samples^37^. The values of replicate probes were averaged, and the data matrix was filtered to 20,000 probes based on the highest median expression values. Clusters of co-expressed genes were identified using K-means clustering (k = 10) on log_2_-transformed and gene-median-centered data with un-centered correlation as a similarity metric in Gene Cluster 3.0 ^38^. Hierarchical clustering was performed using Gene Cluster 3.0, and heat maps were displayed using Java Treeview^39^. Gene Ontology^40^ enrichment analysis was performed using GoMiner^41^. Functional annotation was further analyzed using the Database for Annotation, Visualization, and Integrated Discovery (DAVID) v6.7 ^42^. Transcripts that were significantly associated with the PKR1 phenotype were identified using two-class unpaired Significance Analysis of Microarrays (SAM)^43^. The GEO data link is https://www.ncbi.nlm.nih.gov/geo/query/acc.cgi?acc=GSE94603.

### Western Blot blot analyses

Western blots were performed using 30- µg of total proteins as previously described^44^. The proteins were separated under denaturing conditions by SDS-PAGE (10% gel) and transferred to a polyvinylidene difluoride (PVDF) membrane. The blots were incubated with a blocking solution and then incubated overnight with primary antibodies against peroxisome proliferator-activated receptor gamma (PPARγ, Cell Signaling Technology) at 4 °C with gentle shaking with primary antibodies to PPARγ (cell Signaling Technology). The membrane was washed and then incubated with a horseradish peroxidase-conjugated anti-goat secondary antibody for 1 h at room temperature with gentle shaking. The protein bands were visualized after a 5-min incubation using by enzyme-linked chemiluminescence (GE HealthCare, Piscataway, NJ, USA). The signals were quantified by scanning laser densitometry and normalized to total amounts of the corresponding reference protein expression levels.

### Isolation and culture of the cells

Mice (n = 6, per group) were euthanized by cervical dislocation, and tissues were collected for the isolation of the cardiac cells. To isolate Tomato^+^ cells, tcf21-Cre^TM^ or *PKR1* ^*tcf*−*/*−^ hearts (n = 6) were dissociated to single cells by digestion with 0.1% collagenase IV (Sigma-Aldrich) and 0.05% trypsin (Invitrogen) in Hank’s balanced salt solution (HBSS, (Sigma-Aldrich). Tomato^+^ cells were isolated from tcf21-Cre^TM^ or *PKR1* ^*tcf*−*/*−^ hearts by FACS sorting as previously described^45,46^. The tcf21^+^ cells were cultured in Dulbecco’s modified Eagle’s medium (DMEM), with and without an adipogenic induction cocktails (AIC), containing 1 μM dexamethasone (Sigma), 500 nM 3-isobutyl-1-methylxanthine (IBMX, Sigma) and 10 μg/mL insulin (Sigma) for 7 days at 37 °C under 5% CO_2_^7^. Tcf21^+^ cells were fixed, followed by immunostaining for platelet endothelial cell adhesion molecule 1 (PECAM)-1 and α-SMA or Oil Red O staining were performed. Epicardial explants and culture from TG-PKR1 or WT mice (12w, n = 6) were isolated and cultured as previously described^9^. Cardiomyocytes from 4-week-old mice (n = 6) were isolated by the Percoll gradient technique as previously described^29^. The cardiomyocytes were infected by PKR1 for 48 h, and then the conditioned medium (MM) was collected and concentrated 10-fold using 10-kDa molecular weight (MW) cut-off filter units (Millipore)^47^. Tcf21^+^ cells were then grown in the MM or MM supplemented with AIC with or without prokineticin-2-neutralizing antibody (Bv8, Abcam, 5 ng/ml)^48^. All values are representative of at least three independent experiments.

### Immunostaining

For immunofluorescence assays, frozen tissue sections were fixed, blocked and incubated with primary antibodies against PECAM-1, alpha-smooth muscle actin (α-SMA) and calponin (Sigma); PKR1 (IGBMC, Illkirch); PPARγ and perilipin-A (Cell Signaling Technology); Tomato and Flk-1 (Abcam). Bound antibodies were detected by incubating with fluorescein-, Alexa 555-, Alexa 488- or Alexa 594-conjugated (Millipore) secondary antibodies. Data were analyzed using a Leica fluorescence microscope^49^. Quantification was performed with ImageJ software by counting 20 fields per tissue section at 20x or 40x magnification for each group of mice (at least 3 mice/group) or for each group of cells treated under different conditions.

### Oil Red O staining and lipid isolation

CF-like cells and tcf21^+^ cells were stained with 0.5% Oil Red O solution (Sigma), and visualized under an inverted microscope (Olympus)^7^. Lipid isolation was performed in heart tissue as previously described^7^.

### Tube formation

After treating tcf21^+^ cells for 7 days, the cells were seeded onto 24-well culture plates coated with Matrigel for 24 h (BD Biosciences, Bedford, MA) according to the manufacturer’s instructions. Tube formation was quantified per well as previously described^44^.

### RNA isolation and real-time PCR

Total RNA was prepared from cells or hearts using Tri-Reagent (MRC, Cincinnati, OH) according to the manufacturer’s instructions^9,33^. Total RNA (1 μg) was reverse transcribed with Super Script II Reverse Transcription Reagents (Invitrogen). The resultant cDNA was subjected to real-time quantitative PCR. The primers used for real-time PCR are shown in Table 2. Real-time quantitative PCR was carried out in an iCycler myiQ apparatus (Bio-Rad, Life Science Research, Hercules, CA) using SYBR green fluorescent dye (Bio-Rad). Relative quantification of mRNAs was determined using the ^ΔΔ−^CT method following normalization to glyceraldehyde 3-phosphate dehydrogenase (GAPDH) mRNA and expressed as a fold-change compared to the control^7^.

### Adenovirus infection

Cells were infected with adenoviruses carrying PKR1 cDNA or control cDNA as previously described at a multiplicity of infection of 5^29^.

### Echocardiographic measurements

Mice (14 w) were continuously anesthetized by 1.5–2% isoflurane inhalant to maintain a light sedation level throughout the echocardiographic procedure. Systolic function in male mice (n = 6–8 for each group) was assessed by echocardiography in M-mode and two-dimensional measurements as previously described^9,29^.

### Data analysis and statistics

All experiments were conducted on at least three biological replicates with at least three technical replicates unless otherwise stated. All values are expressed as the mean ± SEM. Statistical analyses were performed using IBM SPSS software (version 20) using either an independent Student’s *t*-test or a multivariate analysis of variance (ANOVA) with Bonferroni *post hoc* analysis. Parameters measured over multiple time points were analyzed by repeated-measure ANOVA with time as a within-subject factor. Statistical significance was considered to be *P* < 0.05.

## Electronic supplementary material

Supplementary Information
