# Supplementary material for: Prokineticin receptor-1-dependent paracrine and autocrine pathways control cardiac tcf21+ fibroblast progenitor cell transformation into adipocytes and vascular cells
Source: Sci Rep. 2017 Oct 16;7:12804. doi: 10.1038/s41598-017-13198-2 (PMC5643307; doi:10.1038/s41598-017-13198-2)
Supplement: Supplementary file 1 — Supplementary Information [file 41598_2017_13198_MOESM1_ESM.pdf]

Supplementary Information

Prokineticin receptor-1-dependent paracrine and autocrine pathways control cardiac tcf21^{+} fibroblast progenitor cell transformation into adipocytes and vascular cells

Rehana Qureshi[1], Michel Kindo[2], Himanshu Arora[1], Mounia Boulberdaa[1], Marja Steenman[3], Canan G. Nebigil[1]

[1] University of Strasbourg, CNRS, Biotechnology and Cell Signaling Laboratory (UMR 7242), Illkirch, France

[2] Hospital of University of Strasbourg, Cardiovascular surgery department, Strasbourg

[3] Institute of Thorax, INSERM UMR 1087 / CNRS UMR 6291, Nantes

FIGURES

Pathway:PPAR signaling pathway

Pathway information generated by KEGG. Stop Blinking

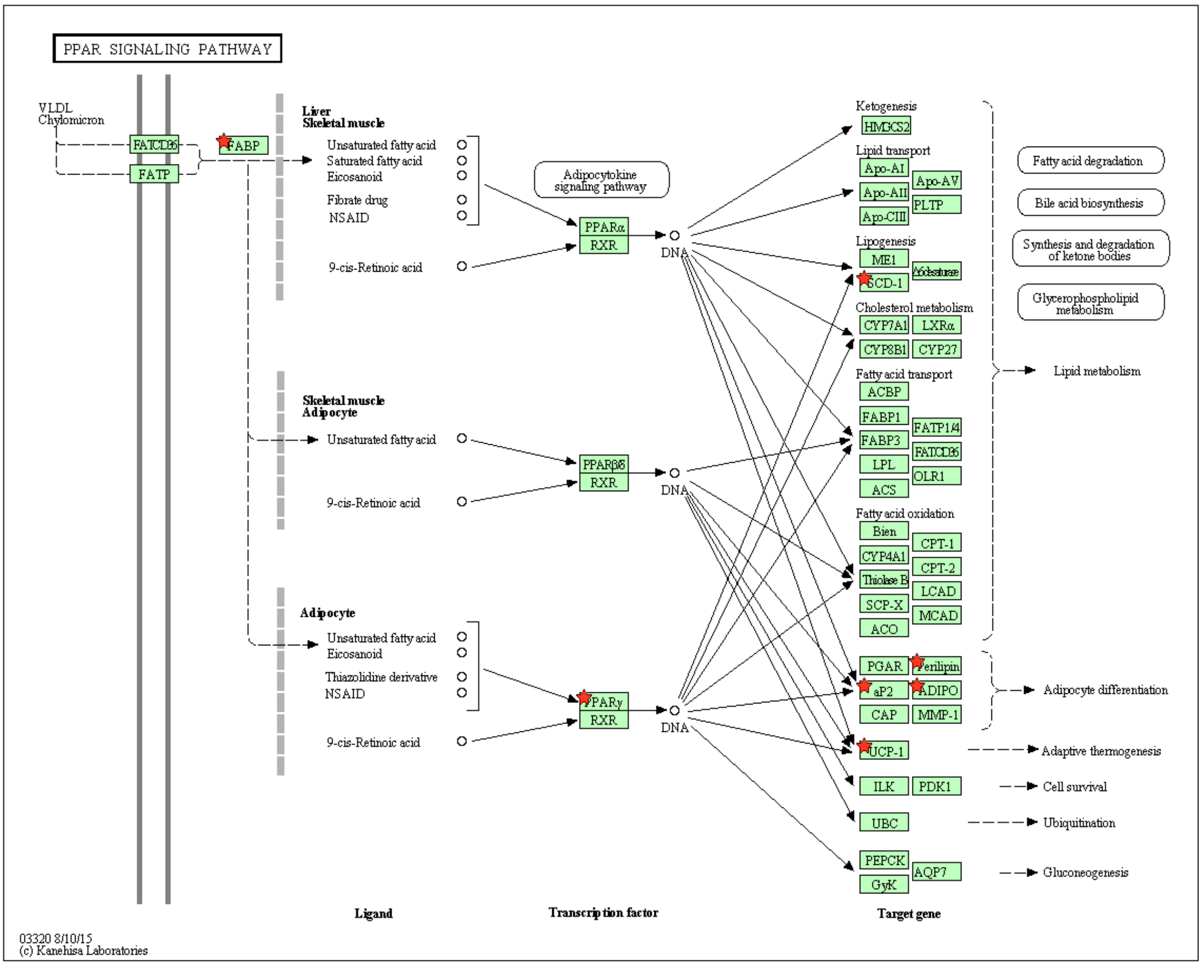

Figure S1. PPARγ signaling pathway analysis generated by KEGG mapping and KEGG pathway image was utilized with a permission of KEGG (http://www.kegg.jp/kegg/kegg1.html), (Kanehisa, M. and Goto, S.; KEGG: Kyoto Encyclopedia of Genes and Genomes. Nucleic Acids Res. 2000 : 28, 27-30). Red stars show the genes that were abolished in TG-PKR1 hearts.

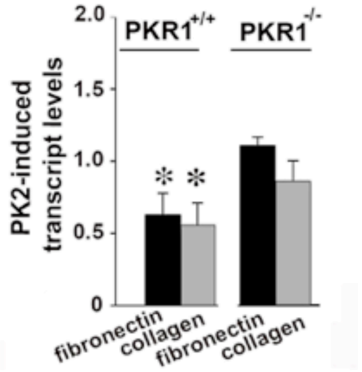

Figure S2. Effect of PKR1 signaling on the fate of tcf21^{+} progenitors**.** Myofibroblast marker expression detected by qPCR in control (PKR1$^{+/+}$) or PKR1 deficient tcf21^{+} (PKR1$^{-/-}$) progenitors upon prokineticin-2 (PK2, 10nM) treatment (*p<0.05, n=5).

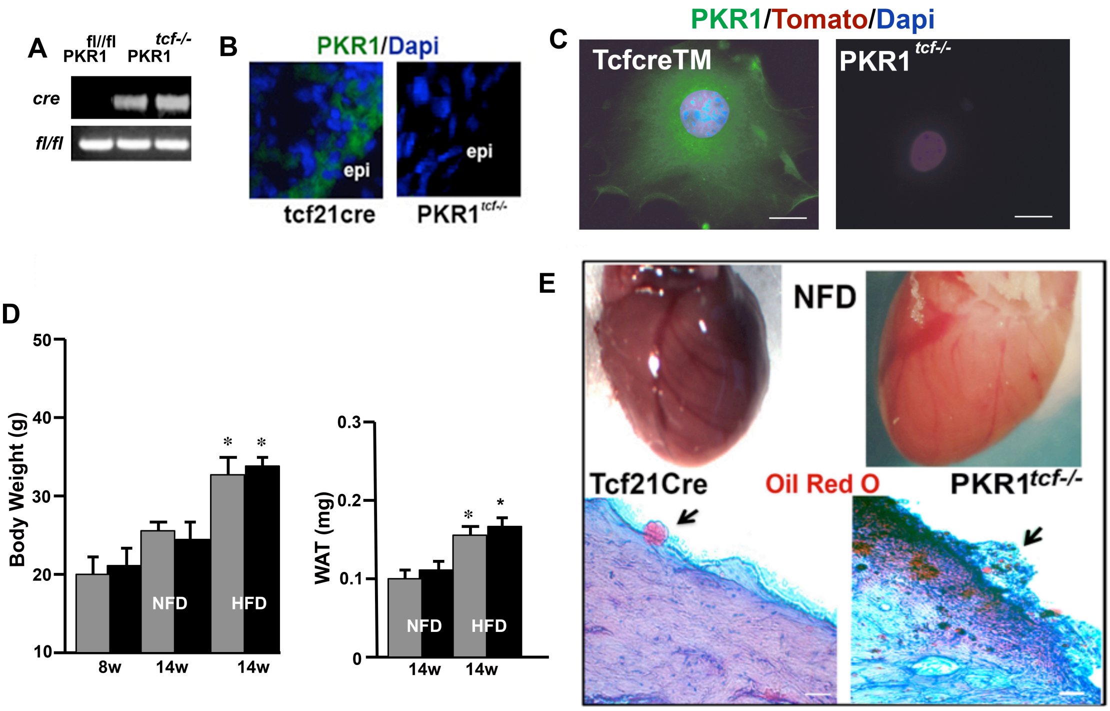

Figure S3. A) Genotyping of mice. Both Cre^{TM+} and PKR1 floxed (fl/fl) mice^{+} lines shows PKR1$^{tcf-/-}$ geneotypes. **B)** PKR1 immunostained hearts. PKR1$^{tcf-/-}$ did not have any PKR1 expression in their epicardial cells (n=6 mice per group). **C)** PKR1 expression in the tomato+ cells FACS isolated after TMX injected tcf21cre^{TM} and PKR1$^{tcf-/-}$ hearts (n=30 cells), confirming no detectable PKR1 protein in mutant tomato+ cells. **D)** Verification of effect of HFD on body morphology. Left histogram shows body weight of 8 weeks old mice and body weights of these mice 4 weeks after NFD and HFD treatments. Right histogram shows WAT weight of two genotypes 4weeks after NFD and HFD exposure. (*p<0.05 as compare to NFD fed mice, n=6 mice per group). **E)** NFD exposed hearts. Gross morphology of hearts and Oil-red-O stained heart sections derived from control and PKR1$^{tcf-/-}$ mice fed a NFD, n=6 mice per group**.**

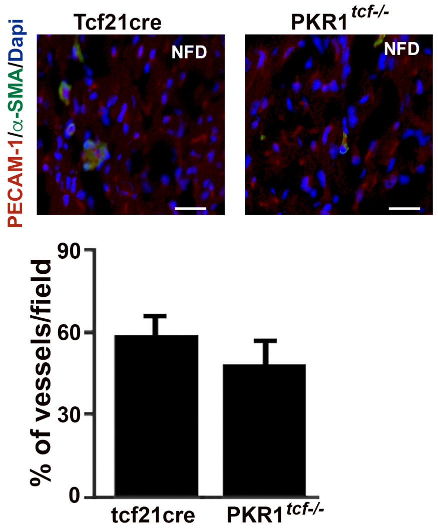

Figure S4. Vascularization of the Non fat diet (NFD) fed mice hearts. Representative of illustration shows PECAM-1 and α-SMA co-stained heart samples derived from tcf21cre (control) and PKR1$^{tcf-/-}$ mice fed a NFD. Histogram shows cardiac vessel numbers are not significantly different between the genotypes (p>0.05, n=5).

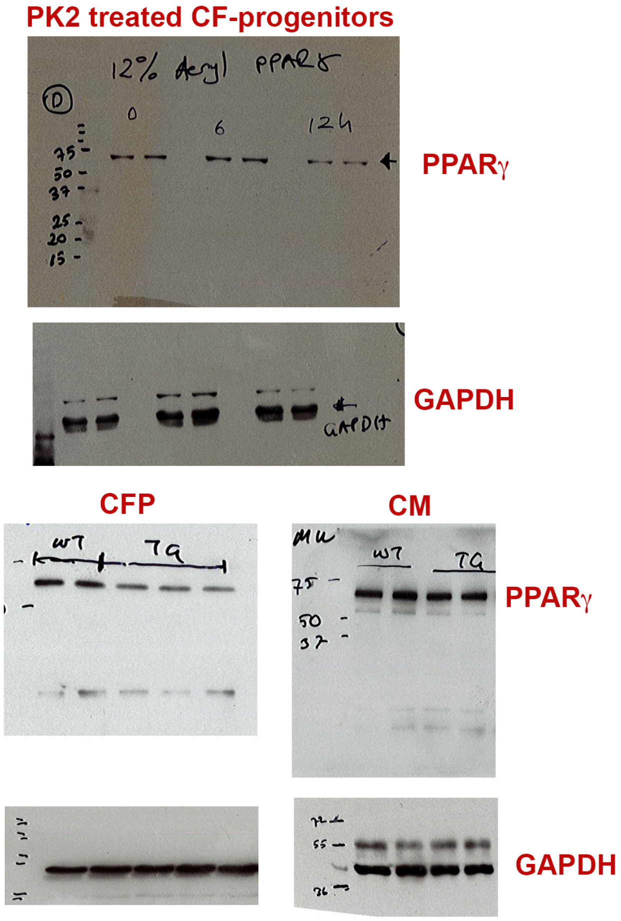

Figure S5. Original blots that were illustrated in Figure 1.
